# Differentiating Memory Care: Regulatory Differences for Memory Care in Assisted Living

**DOI:** 10.1093/geroni/igaf122.4102

**Published:** 2025-12-31

**Authors:** Lindsey Smith, Erh-Chi Hsu, Adam Pennavaria, Kelsey MacKenzie, Cassandra Hua, Jamie Cho, Paula Carder

**Affiliations:** Oregon Health & Science University, Portland, Oregon, United States; Johns Hopkins University, Baltimore, Maryland, United States; Oregon Health & Science University, Portland, Oregon, United States; Oregon Health & Science University, Portland, Oregon, United States; University of Massachusetts Lowell, Lowell, Massachusetts, United States; Johns Hopkins University, Baltimore, Maryland, United States; Portland State University, Portland, Oregon, United States

## Abstract

While prior research has categorized regulatory oversight of AL, we lack a clear understanding of what regulations differ for dementia-specific compared to general AL. Using a health services regulatory analysis of 2023 AL regulations, we examine how dementia care is regulated across the 45 states with dementia-specific AL policies. States most often add memory care-specific rules beyond general AL, including allowing provision of dementia-specific services (18 states), advertising such services (14), requiring controlled egress (13), addressing dementia-related behaviors (13), limiting admission to people living with dementia (12), requiring a dementia-specific activity program (12), mandating dementia-specific staff training where not required in general AL (10), applying certification to only part of a campus (9), requiring a secured outdoor space (9), involving family in care (8), having defined methods for identifying dementia (8), requiring staffing ratios (7), and cognitive impairment screening at admission (5). These requirements highlight substantial variation in how states define and regulate dementia care in AL. Understanding these regulatory differences is important for assessing how they impact care quality, resident safety, and access to memory care—and can inform decision-making for residents, families, and policymakers.

